# From Genome Diversity to Inferred Functional Constraints: An Integrated Evolutionary Analysis of Hepatitis B Virus Genotype F

**DOI:** 10.3390/ijms27052284

**Published:** 2026-02-28

**Authors:** Ruy D. Chacón, Obert Marín-Sánchez, Jimmy Ango-Bedriñana, Homero Ango-Aguilar

**Affiliations:** 1 Department of Pathology, School of Veterinary Medicine, University of São Paulo, Av. Prof. Orlando M. Paiva, 87, São Paulo 05508-270, Brazil; 2 Pathogen Genetics Research Group (PATHO-GEN), OMICS, Lima 15001, Peru; omarins@unmsm.edu.pe; 3 Immunology and Cancer Research Group (IMMUCA), OMICS, Lima 15001, Peru; 4 Departamento Académico de Microbiología Médica, Facultad de Medicina, Universidad Nacional Mayor de San Marcos, Lima 15081, Peru; 5 Instituto de Investigación de Ciencias Biológicas Antonio Raimondi (ICBAR), Facultad de Ciencias Biológicas, Universidad Nacional Mayor de San Marcos, Av. Carlos Germán Amezaga 375, Lima 15081, Peru; 6 Centro de Investigaciones Biocientífica SRL-Clínica El Nazareno-Huamanga, Ayacucho 05001, Peru; jimmy.ango@unsch.edu.pe; 7 Escuela Profesional de Medicina Humana, Universidad Nacional de San Cristóbal de Huamanga, Portal Independencia N° 57–Huamanga, Ayacucho 05001, Peru; 8 Programa Académico de Microbiología, Facultad de Ciencias Biológicas, Universidad Nacional de San Cristóbal de Huamanga, Portal Independencia N° 57–Huamanga, Ayacucho 05001, Peru

**Keywords:** hepatitis B virus, genotype F, molecular evolution, recombination, selective pressure, N-glycosylation, mutations, drug resistance

## Abstract

Hepatitis B virus (HBV) genotype F is one of the most genetically divergent and evolutionarily ancient HBV lineages and predominantly circulates in indigenous and admixed populations of the Americas. Here, we performed a comprehensive evolutionary and inferred functional characterization of the HBV genotype F via the largest curated dataset of complete genomes. Phylogenomic reconstruction, recombination screening, and phylogenetic network analyses were integrated with codon-based selective pressure inference, surface protein posttranslational modification profiling, mutational analysis of antigenic regions, and reverse transcriptase (RT) drug resistance assessment. The HBV-F subgenotype exhibited a well-resolved phylogenetic structure and limited intragenotypic recombination, while intergenotypic recombination contributed substantially to reticulate evolutionary signals. Selective pressure analyses revealed strong purifying selection in replication-associated domains of the polymerase, in contrast to episodic adaptive evolution in surface-exposed and regulatory proteins, particularly the X protein. N-glycosylation sites in large surface proteins are highly conserved. Some mutations in the major hydrophilic region (MHR) were significantly detected, whereas RT drug resistance mutations were rare and followed canonical lamivudine-associated pathways. Collectively, these findings highlight the balance between deep evolutionary conservation and localized adaptive flexibility in shaping the HBV genotype F and provide a genotype-specific framework for interpreting viral fitness, immune interactions, and antiviral resistance.

## 1. Introduction

Hepatitis B (HB) is a viral infection that affects the liver and can cause both acute and chronic disease. It remains a major global public health problem, with an estimated 254 million people infected worldwide (approximately 3.3% of the global population), 1.2 million new infections each year, and more than 1.1 million annual deaths attributable to HBV-related diseases, including cirrhosis and hepatocellular carcinoma (HCC) [1,2,3,4].

The etiological agent, hepatitis B virus (HBV), is a small, enveloped virus belonging to the genus *Orthohepadnavirus* within the family *Hepadnaviridae*. It is characterized by a compact, partially double-stranded DNA genome of approximately 3.2 kb. The viral genome contains four overlapping open reading frames (ORFs) that encode the surface (PreS/S), core (PreC/C), polymerase (Pol), and X (HBx) proteins [5].

Despite its remarkably small genome, hepatitis B virus (HBV) displays extensive genetic diversity. This diversity is driven primarily by the error-prone reverse transcription step during viral replication, which is mediated by a viral polymerase lacking proofreading activity and results in a high mutation rate [6,7]. Additional evolutionary mechanisms, including homologous recombination, immune-mediated selective pressure, and large effective population sizes, further contribute to HBV genetic variability [8,9,10].

On the basis of nucleotide sequence divergence, HBV is currently classified into ten genotypes (A–J), with further subdivision into subgenotypes and phylogenetic clusters. These genotypes are associated with distinct geographic distributions and differ in transmission dynamics, clinical course, response and resistance to antiviral therapies, long-term disease outcomes, and patterns of virulence and pathogenicity [7,11,12,13].

Among the HBV genotypes, genotype F is of particular interest because of its marked genetic divergence and unique evolutionary history. Phylogenetic and phylogeographic studies indicate that genotype F represents one of the most ancient HBV lineages, likely introduced into the Americas through early human migrations and subsequently shaped by long-term host–virus coevolution [14,15,16]. This genotype is predominantly found in indigenous and admixed populations across South America, Central America, and parts of North America, although its geographic distribution also includes documented detections in Europe, Asia, and Oceania, likely reflecting historical and contemporary human mobility [11,16,17,18,19,20,21].

Genotype F is subdivided into six subgenotypes (F1–F6), with additional phylogenetic structuring described within subgenotypes F1 (F1a, F1b, F1c) and F2 (F2a, F2b), reflecting distinct geographic clustering and evolutionary trajectories [20,22]. Clinically, the HBV genotype F has been associated with early-age infection, rapid disease progression, and an increased risk of severe liver disease and hepatocellular carcinoma in certain populations, particularly in South America [23,24,25]. Despite its clinical relevance and evolutionary distinctiveness, genotype F remains underrepresented in integrated large-scale genomic studies, as many previous analyses have relied on limited numbers of sequences or partial genomic regions. This limitation hampers comprehensive inference of its evolutionary dynamics, recombination patterns, and genotype-specific functional constraints.

The generation of mutations in hepatitis B virus (HBV) is highly relevant for understanding the wide spectrum of viral phenotypes and clinical presentations. Although drug resistance mutations have been extensively characterized in HBV genotypes B, C, and D, data on natural polymorphisms and resistance-associated mutations in genotype F remain scarce. This knowledge gap is particularly important because genotype-specific amino acid backgrounds can modulate the genetic barrier to resistance and influence the phenotypic impact of well-established resistance mutations in the viral polymerase [24,26]. In addition, accumulating evidence indicates that posttranslational modifications of HBV proteins play critical roles in regulating viral replication, protein stability, and host–virus interactions; however, these mechanisms have been poorly explored in genotype F [27,28]. Similarly, mutations associated with increased virulence, immune escape, and hepatocarcinogenesis have been investigated predominantly in non-F HBV genotypes [7,29,30]. Taken together, the limited characterization of these functionally relevant elements in genotype F underscores the need for genotype-specific genomic and functional analyses to better elucidate its evolutionary dynamics, clinical behavior, and response to antiviral therapy [7,19,31].

The aim of this study was to provide a comprehensive genomic characterization of the hepatitis B virus (HBV) genotype F via the largest curated dataset of complete genome sequences available to date. By integrating phylogenomic reconstruction with analyses of genetic recombination, selective pressures, posttranslational modifications, mutational profiles, and drug resistance-associated variants, we present an updated and integrative view of the evolutionary landscape of the HBV genotype F and discuss its potential functional and clinical implications.

## 2. Results

### 2.1. Global Phylogenomic Reconstruction of HBV-F Reveals Distinct Regional Distributions of Subgenotypes

**Figure 1:**
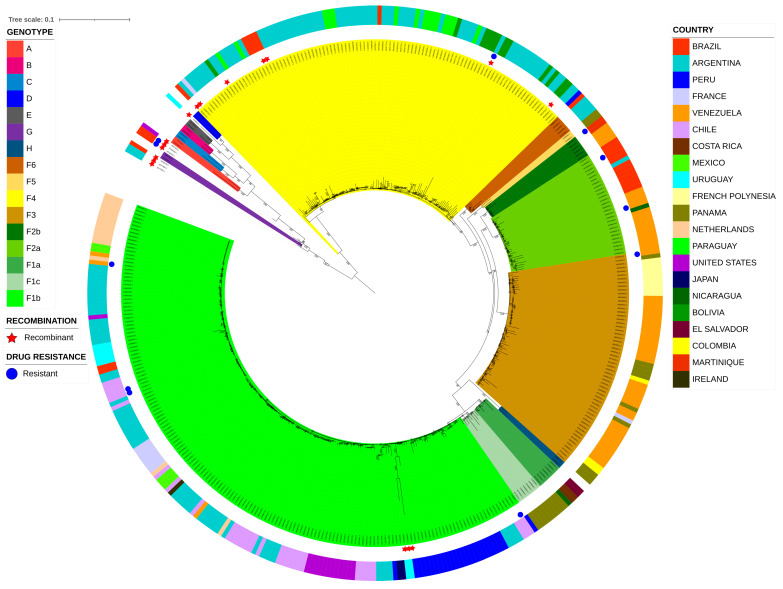
Maximum-likelihood phylogenetic tree of hepatitis B virus (HBV) complete genome sequences. The tree was inferred under a GTR + R substitution model. Branch support was assessed via the approximate likelihood ratio test, with Shimodaira–Hasegawa-like support values (aLRT SH-like) indicated on the branches. The tree scale bar represents 0.1 substitutions per site. The inner colored sectors correspond to HBV genotypes and subgenotypes, as indicated in the legend on the left. The red star symbols denote sequences identified as recombinants. The blue circle symbols indicate sequences carrying drug resistance–associated mutations. The outermost ring represents the country of origin of each sequence, as indicated in the right legend.

For the phylogenetic analysis, the optimal nucleotide substitution model, as determined by the Bayesian information criterion (BIC), was GTR+R, with the proportion of invariable sites fixed. Under this model, a well-resolved phylogenetic distribution of all HBV genotypes (A–H) and HBV-F subgenotypes (F1–F6) was observed (Figure 1).

The most predominant and globally dispersed HBV-F subgenotype was F1b, comprising 165 sequences, which were mainly distributed in South American countries (*n* = 120, 72.7%), including Argentina, Peru, Chile, Venezuela, Uruguay, and Brazil. This was followed by European countries (*n* = 25, 15.2%), including the Netherlands, France, and Ireland; North American countries (*n* = 18, 10.9%), including the United States and Mexico; and a small number of sequences from Asia, represented by Japan (*n* = 2, 1.2%).

The remaining subgenotypes presented clustering patterns driven primarily by geographic proximity and, to a lesser extent, sociocultural links. For example, subgenotype F4, the second most abundantly represented group, consisted almost exclusively of sequences from the southern cone of South America (*n* = 100, 98.1%), including genomes from Argentina, Paraguay, Bolivia, Brazil, and Peru, with only one genome from France and one from Martinique. Subgenotype F3, the third most abundant group, was composed mainly of sequences from the northern cone of South America (*n* = 38, 63.3%), predominantly from Venezuela, and to a lesser extent from Colombia and Peru, as well as from Panama in Central America (*n* = 11, 18.3%), a country that borders Colombia. In addition, one genome from France and ten from French Polynesia were identified, suggesting a sociocultural association.

Similarly, subgenotype F2a, which is phylogenetically close to F3, was also dominated by sequences from northern South America (*n* = 25, 92.6%), mainly from Venezuela and Brazil, with only one genome from Argentina and two additional genomes from Nicaragua in Central America. Although the remaining subgenotypes were less frequently represented, they largely retained these geographic trends. Subgenotype F1a (*n* = 7), for instance, included exclusively Central American genomes from Costa Rica, El Salvador, Panama, and Nicaragua. Similarly, subgenotypes F1c and F5 were restricted to genomes from Panama (*n* = 7 and *n* = 2, respectively). Subgenotype F2b (*n* = 6) included genomes from Venezuela and Martinique, a Caribbean island in close geographic proximity to Venezuela, whereas subgenotype F6 (*n* = 5) was restricted to genomes from Argentina and a single genome from Brazil.

**Figure 2:**
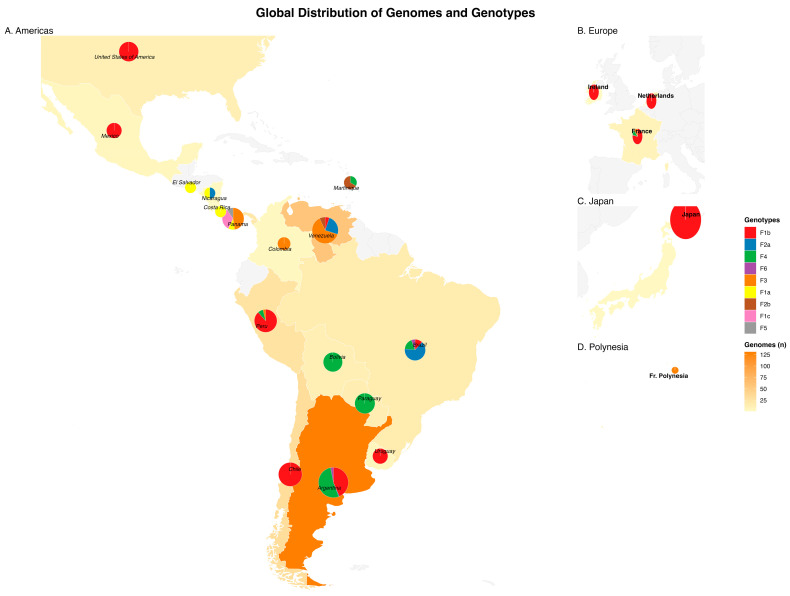
Global geographic distribution of hepatitis B virus (HBV) genotype F complete genome sequences. Maps are shown for (**A**) the Americas, (**B**) Europe, (**C**) Asia (Japan), and (**D**) Oceania (French Polynesia). Countries are shaded according to the total number of genomes analyzed and available per location, as indicated by the color scale (genomes, n). Pie charts positioned over each country represent the relative proportion of the HBV genotype F subgenotype (F1a, F1b, F1c, F2a, F2b, F3, F4, F5, and F6), with colors defined in the legend. The size of each pie chart is proportional to the number of genomes available for that country.

In agreement with the phylogenetic patterns shown in Figure 1, geographic mapping of the HBV genotype F genomes revealed marked spatial heterogeneity in both genome abundance and subgenotype composition across regions (Figure 2). The highest concentration of genomes was observed in South America, particularly in southern Cone and northern South American countries, which also presented the greatest subgenotype diversity. In contrast, Central American countries presented a more restricted distribution, with fewer genomes and a limited number of subgenotypes, yet clear regional specificity was retained. However, it is important to note that the geographic representation of genomes in public databases is influenced by data availability. Consequently, the observed subgenotype proportions should be interpreted with caution in countries with small sample sizes, where estimates of relative frequency may be unstable. In this context, Figure 2 also provides country-level genome counts through both color shading and proportional pie chart size to facilitate interpretation.

In addition to the American continent, the map highlights the presence of the HBV genotype F in Europe, Asia, and Oceania, where detections are sporadic and generally characterized by reduced subgenotype diversity. These extracontinental occurrences were predominantly associated with a limited number of countries, including France, the Netherlands, Ireland, Japan, and French Polynesia, underscoring the broad geographic reach of genotype F despite its recognized American origin.

### 2.2. Recombination and Network Analyses Reveal Intergenotypic Recombination and Intragenotypic Structuring in HBV-F

#### 2.2.1. Recombination Analyses

Genome-wide recombination analysis identified 13 potential recombination events involving 17 recombinant strains with genetic contributions from HBV genotype F (Table 1 and Table S2). This corresponds to 4.30% of the total number of HBV genotype F genomes analyzed (*n* = 395). In ten of these strains, genotype F acted as the major parental lineage, whereas in the remaining cases, it contributed as a minor parent. The most frequent parental genotype combination was F/G (*n* = 7), followed by F/D (*n* = 4). HBV genotype G was the most commonly detected recombination partner with HBV-F (*n* = 9), whereas the other genotypes involved included D (*n* = 5), A (n = 4), H (*n* = 2), and B (*n* = 1). No recombinant strains involving HBV genotypes C or E were identified.

Four recombinant strains presented evidence of multiple parental lineages, including three strains with three potential parental genotypes and one strain with four. Notably, only a single recombinant strain (MG098579) presented a significant signal of intragenotype recombination within HBV-F.

#### 2.2.2. Phylogenetic Network Analyses

**Figure 3:**
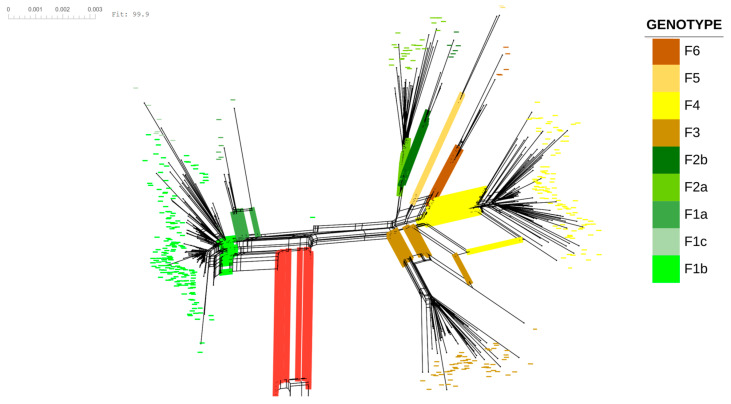
Neighbor-Net phylogenetic network of hepatitis B virus (HBV) genotype F complete genomes. The network depicts nontree-like evolutionary relationships among HBV genotype F sequences inferred from pairwise genetic distances. The terminal nodes represent individual genomes, whereas the edges and parallelogram-like structures indicate conflicting phylogenetic signals that are consistent with reticulate evolution. The sequences are grouped according to genotype F subgenotypes, as indicated by the color-coded background in the legend. The nodes, branches, and background highlighted in red correspond to three recombinant genomes included as internal controls, illustrating their contributions to reticulate signals and network complexity. The scale bar represents genetic distance (substitutions per site).

The phylogenetic network of the HBV-F genomes revealed clear and well-defined structuring among the subgenotypes (Figure 3), which was consistent with the phylogenomic analyses described in Section 2.1. This pattern was independently supported by principal coordinate analysis (PCoA; Figure S1), in which subgenotype-specific clusters were clearly separated in multidimensional genetic space. Within the network, each subgenotype formed a largely coherent cluster represented by distinct colors, reflecting strong genetic differentiation and internal cohesion.

Network statistics revealed low levels of reticulation within HBV-F. The delta score (−0.612600) and Q-residual score (−0.007452) were indicative of a predominantly tree-like signal, which is consistent with limited topological conflict among subgenotypes. In agreement with this pattern, the Phi test for recombination did not detect a significant recombination signal within the HBV-F dataset (*p* = 1). Reticulations observed among closely related subgenotypes, particularly between F3 and F4, were sparse and were restricted mainly to internal branches, suggesting shared ancestral variation or incomplete lineage sorting rather than detectable intragenotype recombination; in contrast, the three recombinant control strains included in the analysis already displayed markedly reticulate connections under this restricted dataset.

In contrast, the inclusion of recombinant sequences and genomes from HBV genotypes other than F (*n* = 409 genomes) resulted in markedly different network topologies. Under this expanded dataset, the delta score (2.235000) and the Q-residual score (0.043650) indicated substantially increased reticulation and topological conflict, reflected by pronounced reticulate patterns and divergent connections within the network. Consistently, the Phi test strongly supported the presence of recombination (*p* = 4.421 × 10^−9^), corroborating the detection of intergenotypic recombination events in HBV. Together, these results indicate that the recombination signal observed in the combined dataset is driven primarily by intergenotypic recombination, whereas the HBV-F subgenotypes retain a largely tree-like evolutionary structure once the recombinant and non-F genomes are excluded.

### 2.3. Selection Pressure Analysis Reveals Contrasting Evolutionary Regimes Across HBV-F Proteins

**Figure 4:**
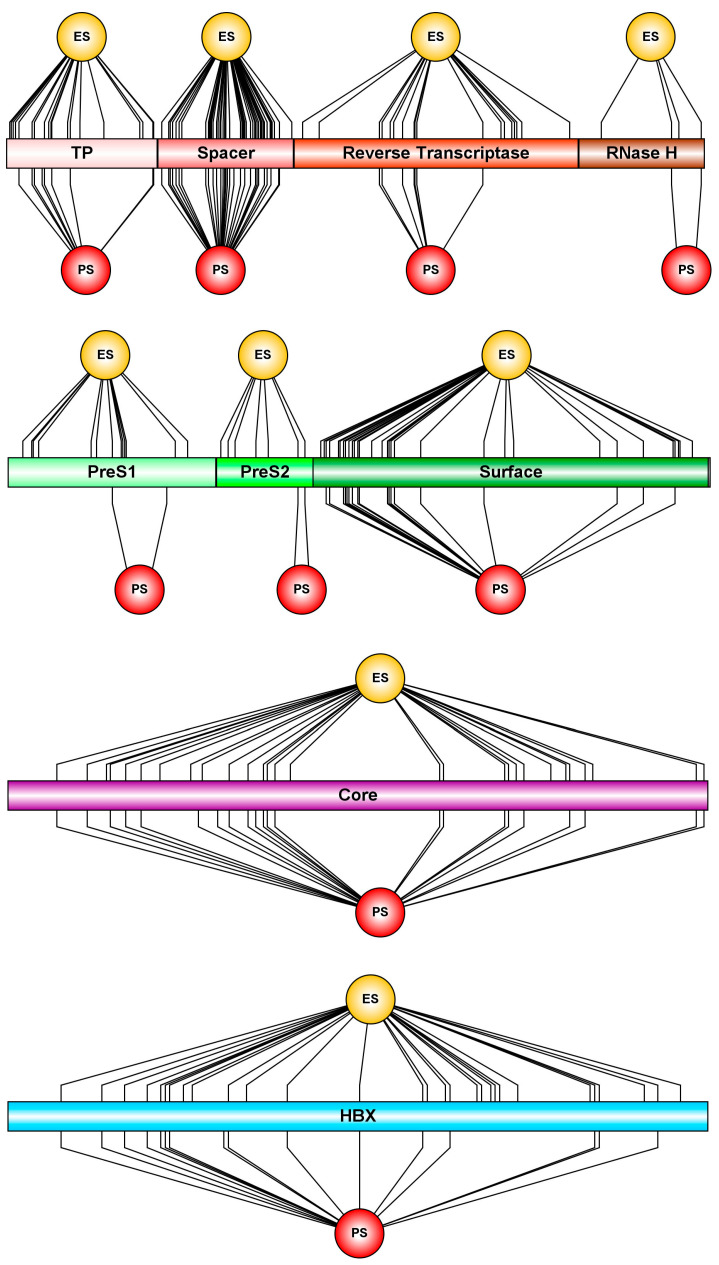
Schematic representation of HBV proteins highlighting codons under positive selection. Linear diagrams of the HBV polymerase, surface, core, and X proteins were generated, with functional domains indicated along each protein sequence. Codon sites inferred to be under episodic positive selection (ES) are marked by lines converging toward a yellow sphere. Codon sites identified as being under pervasive positive selection (PS) are indicated by lines converging toward a red sphere.

Analyses of selective pressure across the HBV-F proteome revealed a heterogeneous distribution of episodic, pervasive positive, and negative selection among viral proteins and functional domains (Table 2 and Table S3, Figure 4). Overall, negative (purifying) selection predominated in structurally and enzymatically constrained regions, particularly within the polymerase protein. The reverse transcriptase (RT) and RNase H domains presented high densities of negatively selected codons (0.35 in both domains) but relatively low densities of episodic and pervasive positive selection. Similarly, the terminal protein (TP) domain displayed a strong signal of purifying selection (density = 0.42), which is consistent with the functional constraints associated with viral replication. In contrast, the spacer domain of the polymerase showed a markedly different pattern, with elevated densities of episodic (0.28) and pervasive (0.18) positive selection and comparatively low negative selection (0.07), indicating relaxed functional constraints and increased evolutionary flexibility in this region.

Distinct selective regimens were also observed among the structural and regulatory proteins. The surface protein (S) exhibited moderate but consistent signals of episodic and pervasive positive selection across PreS1, PreS2, and S domains, accompanied by lower densities of negative selection relative to polymerase domains. The core protein (C) showed a mixed pattern, with comparable densities of episodic (0.15) and pervasive (0.12) positive selection alongside a substantial signal of purifying selection (0.34). Notably, the X protein displayed the most pronounced signal of episodic positive selection across the HBV-F genome, with more than half of its codons under episodic selection (density = 0.53) and relatively low densities of negative and pervasive selection. Collectively, these patterns indicate that HBV-F evolution is dominated by strong purifying selection in replication-associated proteins, in contrast to episodic adaptive diversification in regulatory and surface-exposed proteins, particularly the X protein and nonenzymatic regions of the polymerase.

### 2.4. N-Glycosylation Mapping Reveals a Conserved Structural Framework and Immune-Relevant Variability in the HBsAg MHR

N-glycosylation analysis identified sequons at 1796 sites, of which 732 corresponded to the NXS motif and 1064 to the NXT motif. The genomes exhibited variability in the number of potential N-glycosylation sites, with 3 sites in 0.83% (3/362), 4 sites in 9.12% (33/362), 5 sites in 83.15% (301/362), and 6 sites in 6.90% (25/362) of the sequences. These sites were distributed across the three subdomains of L-HBsAg, with three located in the PreS1-exclusive domain (positions 15, 37, and 46), one in the PreS2-exclusive domain (position 123), and four in the S domain (Table 3).

**Figure 5:**
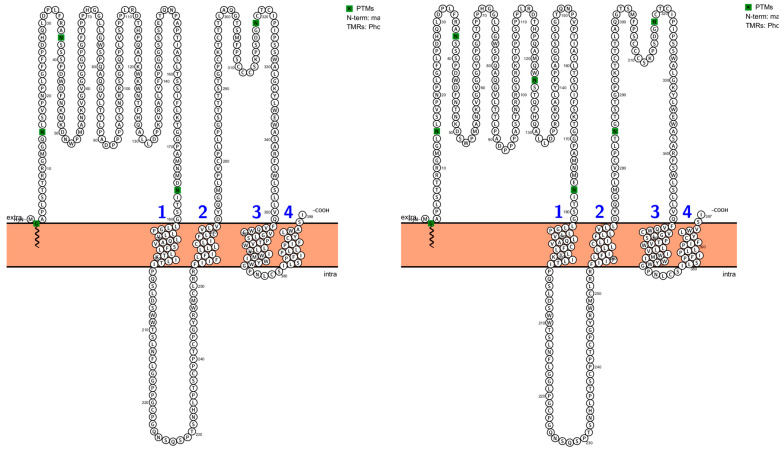
Topology and glycosylation sites of the hepatitis B virus surface antigen (L-HBsAg). **Left panel**: Schematic topology of the HBV L-HBsAg variant without the N123 site (UniProt ID: A0A076L629). **Right panel**: Schematic topology of the HBV L-HBsAg variant with the extra N285 site (UniProt ID: Q9DH30). The model shows the transmembrane organization of the protein across the endoplasmic reticulum membrane, highlighting the extracellular loops and luminal domains. The major hydrophilic region (MHR), including the antigenic determinant loop (“a” determinant), is located in the luminal/extracellular region. The predicted N-glycosylation sites are indicated in green, and the transmembrane helices are labeled numerically. Both diagrams were generated on the basis of their respective UniProt annotations.

Within the S domain, site 177 is located in an extravirion region under the external topology, and sites 285 and 320 are positioned in the extravirion portion of the major hydrophilic region (MHR), which lies between transmembrane regions TM2 and TM3, and one site is located within TM4 (considered nonviable for glycosylation). Sites with extensive experimental evidence of N-glycosylation (positions 15, 123, and 320) were fully conserved (100%), except for ten sequences lacking the motif at position 123 (fraction 0.972), corresponding to the genomes of subgenotypes F1b, F4, and F1c (Figure 5).

Notably, the emergence of an additional motif within the MHR at position 285 was detected in three genomes belonging to subgenotype F3 derived from Yucpa indigenous individuals from Venezuela (GenBank: AB036905–AB036907). Finally, analysis of the N-terminal myristoylation of the surface protein revealed a fully conserved glycine residue at position 2, which was consistent with a 100% conservation rate.

Conversely, the MHR harbored several mutations previously associated with occult hepatitis B infection (OBI) or HBV reactivation (Table 4). Of these, six mutations were located in the N-terminal region of the MHR, with L110I being the most prominent, detected in 25.97% of the sequences. Three mutations were identified within the a″ determinant region, with T140S standing out because of its high prevalence (93.09%). Finally, two highly frequent mutations were detected in the C-terminal region of the MHR, namely, F161Y and V164E, each present in 99.17% of the sequences.

### 2.5. Analysis of Antiviral Resistance Reveals the Restricted Distribution of RT Resistance-Associated Mutations

Drug resistance analysis of the HBV reverse transcriptase identified resistance-associated mutations in 11 out of 362 genomes (3.04%). All resistant sequences carried substitutions affecting the YMDD motif region, predominantly rtM204V/I, frequently accompanied by the compensatory mutation rtL180M (Table 5). Notably, a three-mutation pattern involving rtV173L-rtL180M-rtM204V was detected in one of the analyzed genomes.

The resistance profiles were consistent across nucleos(t)ide analogs, with lamivudine and telbivudine classified as resistant (R), whereas entecavir resistance was predominantly classified as intermediate (I).

Resistant genomes were detected across multiple subgenotypes (F1b, F2a, F2b, and F3) and geographic origins. Importantly, resistance-associated mutations were also identified in three recombinant genomes (Figure 1), suggesting that recombination may contribute to the dissemination or persistence of drug-resistant variants.

## 3. Discussion

HBV genotype F represents one of the most genetically divergent and evolutionarily ancient HBV lineages, with a unique geographic and population-specific distribution in the Americas. In this context, updated genomic studies are essential for monitoring ongoing evolutionary changes and capturing emerging patterns of diversification in a contemporary epidemiological landscape. In this study, we addressed this need by integrating phylogenomic, evolutionary, and computational functional analyses to contextualize how conservation and adaptive variation could shape the biology of the HBV genotype F. Because this study is based primarily on comparative genomic and evolutionary analyses, functional interpretations of mutations should be understood as biologically informed inferences supported by previously reported experimental studies in HBV or related genotypes rather than direct functional validation within genotype F.

The phylogenomic architecture of HBV genotype F reveals a diversification history deeply rooted in the American continent, where the pronounced spatial heterogeneity of its subgenotypes acts as a biological record of past and ongoing human population movements [22,32]. Among these, subgenotype F1b has emerged as the most prevalent and geographically widespread lineage within genotype F, corroborating the prevalence patterns and dispersal trends reported over previous decades [20,32,33,34]. Notably, F1b appears to have overcome its original geographic confinement in the Americas, establishing a cosmopolitan distribution across Europe, Asia, and Oceania, likely driven by a combination of increased viral fitness and its association with large-scale human migration flows [17,18,35,36,37]. In contrast, the remaining subgenotypes display strong regional endemism within the Americas. Clear subregional phylogeographic clustering is observed, with subgenotypes F1a, F1c, F2, F3, and F5 predominantly associated with the northern cones of South America and Central America, whereas subgenotypes F4 and F6 are more closely linked to the southern cone of South America [32,33,38,39]. Within this framework, certain countries emerge as viral biodiversity hotspots, harboring unique subgenotypes such as F1c and F5 in Panama and F6 in Argentina, which are absent from other regions. Together with the presence of more broadly distributed subgenotypes, these patterns position Central and South America as key reservoirs for understanding the ancestral divergence and evolutionary history of the HBV genotype F [39,40,41,42]. Although many of these phylogeographic trends have been previously reported, the present findings reinforce and refine this evolutionary framework via an updated and expanded genomic dataset [15,20,32]. Finally, the detection of specific lineages in geographically distant regions, such as subgenotype F3 in French Polynesia, highlights that sociocultural and colonial links can act as predictors of viral dispersal that are as influential as geographic proximity [21,43]. This overall stability of endemic niches, in contrast with sporadic transcontinental detection, raises important questions regarding HBV adaptive evolution and its long-term coevolution with indigenous and admixed human populations [16,20,42]. A limitation of this study is related to the uneven geographic distribution of publicly available genomes. Sequence repositories often reflect research intensity rather than true epidemiological prevalence, which may influence apparent subgenotype frequencies, recombination detection rates, and the identification of rare mutations in underrepresented regions. Therefore, geographic interpretations should be considered exploratory and hypothesis-generating, pending confirmation from more systematically sampled populations.

HBV has exhibited a gradually increasing level of genetic diversification over recent decades, despite the implementation of global public health measures, including widespread, although still imperfect, access to vaccination programs [44,45]. Extrinsically, HBV evolution is shaped by host–virus interactions operating under selective pressure, as well as by geographic barriers; intrinsically, it is driven primarily by evolutionary forces such as mutation and recombination rates [6,7,8,9,10,44,46,47]. In the present study, 4.30% of the HBV genotype F–associated genomes were identified as potential recombinants. Although this proportion is lower than that reported for more globally distributed genotypes, it nonetheless represents a biologically relevant evolutionary force [46,47]. In descending order, the genotypes most frequently acting as recombination partners of HBV-F were G, D, A, and H, whereas no recombinants involving genotypes C or E were detected. These patterns reflect the heterogeneity of intergenotypic recombination and show a strong, albeit expected, association with geographic proximity or colocalization of circulating genotypes [46,47]. Notably, evidence of supernumerary recombination events involving three or four different genotypes was also detected, suggesting a sequential process in the generation of recombinant strains [46,48]. In contrast, intragenotypic recombination within genotype F was detected only once, constituting a rare finding that required a complementary analytical approach. Accordingly, phylogenetic network analyses were performed after excluding recombinant sequences and non-F genotypes to assess population structure and residual recombination signals. These analyses corroborated previous results, revealing a well-defined subgenotypic structure of HBV-F characterized by a predominantly tree-like topology, lacking topological conflicts or reticulation patterns, and showing no significant evidence of recombination, as supported by the Phi test. This complex and sometimes uneven contribution of recombination may confer biological advantages or reflect recurrent coinfection events, ultimately facilitating the emergence of drug resistance, immune escape during primary infection or vaccination, diagnostic and genotyping failures, and, consequently, contributing to the continued expansion of the HBV epidemic [48,49].

Selective pressure is a central evolutionary force shaping HBV genetic diversity, governing the balance between functional constraints and adaptive flexibility across viral proteins. In HBV, differences in selective regimes among proteins and domains reflect their distinct structural, enzymatic, regulatory, and immunological roles within the viral life cycle [50,51]. In this study, the HBx protein presented a predominance of codons inferred to be under positive selection relative to negative selection, aligning with previous reports detecting signals of adaptive evolution in the HBx region [51,52]. This pattern likely reflects the multifunctional regulatory role of HBx in transcriptional activation, replication control, and host–virus interactions, where adaptive substitutions may increase viral persistence or immune modulation, which is consistent with the functional roles previously reported for HBx in HBV infection [53,54]. In contrast, the core protein presented a greater proportion of codons under negative selection than under positive selection, in agreement with studies showing strong purifying selection acting on capsid-forming proteins [8,55]. This dominance of negative selection is expected given the stringent structural constraints required for capsid assembly, genome encapsidation, and virion stability, where most amino acid changes are deleterious [55,56]. Similarly, the polymerase protein as a whole showed a marked excess of negatively selected sites, reflecting the high functional constraint imposed on its enzymatic domains, including reverse transcriptase and RNase H [57,58]. However, this trend was reversed in the spacer domain, which presented approximately four times more positively selected sites than negatively selected sites, which is consistent with its recognized structural flexibility and tolerance for amino acid variation [57,58]. The spacer domain has been proposed as an adaptive region capable of accommodating immune-driven variation or compensatory changes without compromising polymerase function [58,59]. In the surface protein, selection patterns were balanced, with comparable numbers of positively and negatively selected sites across domains, supporting previous observations that immune-exposed regions such as the major hydrophilic region undergo adaptive diversification, whereas transmembrane segments remain under strong purifying selection [60,61,62]. While our interpretation relies primarily on topological mapping of the surface protein, integration with available or predicted three-dimensional structural models could further refine the spatial context of positively selected residues, MHR variability, and glycosylation motifs. Taken together, these findings highlight a gradient of evolutionary flexibility across the HBV proteome, where regulatory and intrinsically disordered regions accumulate adaptive changes more readily than enzymatic or structural components do [52]. Overall, HBV evolution appears to be largely influenced primarily by mutations in regulatory and immune-exposed domains, whereas enzymatic and transmembrane regions remain evolutionarily conserved, reflecting a trade-off between functional integrity and adaptive potential that underpins viral persistence and diversification [50,52].

The analysis of posttranslational modifications in the hepatitis B virus surface antigen (L-HBsAg) of genotype F is consistent with a molecular architecture that balances structural stability with localized immune evasion strategies on the basis of mechanisms described in other HBV genotypes. The absolute conservation of N-glycosylation sites at positions 15 and 320, together with the 97% preservation observed at position 123, underscores the importance of these carbohydrate moieties for proper protein folding and efficient secretion of viral particles across HBV-F lineages [27,28,63,64,65]. However, the emergence of an additional glycosylation motif at position 285 within the major hydrophilic region (MHR) in subgenotype F3 strains isolated from the Yucpa indigenous community suggests a lineage-specific adaptive mechanism that may favor escape from neutralizing antibodies through masking critical epitopes [66]. Moreover, variability in glycosylation density points to a phenotypic modulation of immune visibility that differs among subgenotypes F1b, F1c, F3, and F4 [64,67,68,69,70,71]. Protein language model–based approaches may also offer complementary perspectives on mutational constraints across overlapping HBV reading frames and represent an interesting direction for future investigations. In contrast to this envelope plasticity, the complete conservation of the N-terminal glycine at position 2, which is required for myristoylation, suggests that the fundamental mechanism of viral entry via the NTCP receptor represents a functionally invariant constraint for infectivity across all HBV-F variants, although direct experimental evidence for these effects in genotype F remains limited [72,73,74].

The major hydrophilic region (MHR) of HBV-F has a significant prevalence of mutations traditionally linked to occult hepatitis B infection (OBI) and viral reactivation, suggesting an intrinsic molecular signature for immune persistence. Specifically, the near-fixation of substitutions such as T140S (93.09%), F161Y (99.17%), and V164E (99.17%) within the ‘a’ determinant suggests a significant divergence in antigenicity that could impair serological HBsAg detection [69,75,76,77,78]. Furthermore, the strategic distribution of these mutations, ranging from the N-terminal (L110I) to the C-terminal domains, may reflect evolutionary patterns that could influence the performance of current diagnostic assays and vaccine-induced antibodies in regions where genotype F is endemic [79,80,81].

The analysis of the HBV-F reverse transcriptase revealed a low but clinically relevant frequency of drug resistance (3.04%), predominantly characterized by substitutions within the highly conserved YMDD motif, which have been experimentally characterized in HBV polymerase across multiple genotypes. The dominance of the rtM204V/I mutation, which frequently cooccurs with the compensatory rtL180M mutation, establishes a robust resistance profile against first-generation nucleos(t)ide analogs such as lamivudine and telbivudine while conferring intermediate resistance to entecavir, as previously reported [82,83]. In addition to simple point mutations, the identification of these resistance-associated mutations (RAMs) in three recombinant genomes across various subgenotypes (F1b, F2a, F2b, and F3) suggests a dynamic evolutionary strategy. This finding implies that recombination may serve as a critical mechanism for the interlineage dissemination of resistance, potentially allowing these stable, resistant variants to persist and circulate even in the absence of direct selective pressure from antiviral therapy [46,84].

## 4. Materials and Methods

### 4.1. HBV Data Set Preparation

The dataset used in this study was assembled with the aim of including all publicly available complete hepatitis B virus (HBV) genomes belonging to genotype F available to date. As an initial framework, all reference genomes corresponding to HBV genotypes A–H as defined by the specialized hepatitis B virus database (HBVdb) were included [85].

To comprehensively identify HBV genotype F sequences, a systematic search strategy was implemented via the Basic Local Alignment Search Tool (BLAST + 2.17.0; https://blast.ncbi.nlm.nih.gov/Blast.cgi, accessed on 12 December 2025). Two full-length HBV genotype F reference genomes (GenBank accession numbers AY090458 and X75658) were used as query sequences. Candidate sequences were retained on the basis of the following inclusion criteria: (i) nucleotide sequence identity greater than 73% and (ii) a minimum genome length exceeding 3000 base pairs. The identity threshold was empirically defined through preliminary screening analyses, which revealed that sequences exhibiting lower identity values consistently detected non-F genotypes.

The dataset was subsequently curated by excluding (i) duplicate genomes, (ii) artificial or laboratory-derived clones, (iii) sequences containing more than 1% ambiguous nucleotides (Ns), and (iv) genomes not assigned to genotype F according to the HBV Sequence Genotyping Tool implemented in HBVdb.

The final dataset consisted of 393 complete HBV genotype F genomes, together with 16 reference genomes, yielding a total of 409 sequences. The complete list of genomes included in the study is provided in Table S1.

### 4.2. Sequence Alignment and Phylogenomic Analysis

All HBV genome sequences were aligned via the online implementation of MAFFT version 7 [86], which applies default parameters and enables the option “adjust direction according to the first sequence”. The resulting multiple sequence alignment was inspected and curated via Geneious Prime^®^ version 2025.1.3 (Biomatters Ltd., Auckland, New Zealand).

Phylogenetic inference was performed via the maximum likelihood (ML) method implemented in PhyML [87]. The optimal nucleotide substitution model was selected on the basis of the Bayesian information criterion (BIC). Branch support was assessed via the approximate likelihood ratio test with Shimodaira–Hasegawa-like support values (aLRT SH-like).

The inferred phylogenetic tree was visualized and edited via the Interactive Tree of Life (iTOL) platform, version 7.4 [88]. Sequence-associated metadata, including country of origin, were graphically annotated onto the tree to facilitate phylogeographic interpretation.

### 4.3. Recombination and Phylogenetic Network Analyses

Genetic recombination analyses were performed via multiple sequence alignment comprising all the genomes included in the phylogenetic analysis (*n* = 409). Recombination detection was conducted via RDP4 version 4.101 [89]. The following seven methods implemented in the software were applied: RDP, GENECONV, BootScan, MaxChi, Chimera, SiScan, and 3Seq.

A genome was considered a putative recombinant only when recombination signals were concordantly detected by at least five of the seven methods and supported by a *p* value < 0.05. When a potential recombinant genome was associated with multiple recombination events, the event showing the strongest statistical support was retained for downstream analyses.

Additionally, phylogenetic network analyses were conducted via SplitsTree App version 6.0.0 [90]. Following recombination screening performed on the complete dataset of 409 genomes, network analyses were carried out via a curated subset of 381 complete HBV genomes. This subset exclusively comprised HBV genotype F sequences, including three genomes previously identified as recombinants by RDP4, which were retained as internal controls to assess the behavior of reticulate signals in the network framework. Pairwise genetic distances were calculated via the p-distance method.

Phylogenetic network reconstruction was performed via the Neighbor-Net algorithm [91]. To statistically evaluate the presence of nontree-like evolutionary signals, the delta score and Q-residual score were computed. In addition, evidence for recombination was assessed via the Phi test for recombination. To further test the hypotheses evaluated by the aforementioned analyses, phylogenetic network analyses were also performed using the original dataset of 409 genomes.

### 4.4. Selection Pressure Analysis

Selective pressure analyses were conducted exclusively on nonrecombinant hepatitis B virus (HBV) genotype F genomes via the Datamonkey web server [92]. Analyses were performed separately for the four HBV open reading frames (ORFs): surface (S), polymerase (P), core (C), and X (X protein). Coding regions corresponding to each ORF were extracted from the whole-genome alignments, and sequences containing inappropriate stop codons were excluded prior to downstream analyses.

To detect episodic positive selection at individual codon sites, the MEME (mixed effects model of evolution) method was applied with a *p* value threshold of 0.1 [93]. In addition, pervasive selection was assessed via FUBAR (fast unconstrained Bayesian approximation), with sites considered significant when supported by a posterior probability ≥ 0.9 [94]. Similarly, the SLAC (Single-Likelihood Ancestor Counting) method was employed with a *p* value threshold of 0.1 to identify codons under pervasive selection [95].

### 4.5. Posttranslational Modification and Antigenic Determinant Analysis of the Surface Protein

In silico predictions of posttranslational modifications (PTMs) were performed on proteins encoded by all nonrecombinant hepatitis B virus (HBV) genotype F genomes, excluding sequences previously identified as recombinant. Only PTM types that have been experimentally reported in prior HBV studies were considered [27].

For surface (S) proteins, N-linked glycosylation sites were predicted via the N-Glycosite [96] by identifying the canonical sequon, which is defined as an oligosaccharide chain attached to asparagine (N) within the tripeptide motif N-X-S or N-X-T, where X represents any amino acid except proline. In addition, the conservation of N-terminal myristoylation was evaluated via the myistoylator tool available through ExPASy [97].

Finally, graphical representations of viral proteins, functional domains, and predicted PTM sites were generated via Protter version 1.0 [98] and IBS 1.0 [99].

### 4.6. Drug Resistance of the Reverse Transcriptase (RT) Domain

Antiviral drug resistance in hepatitis B virus (HBV) was assessed via the specialized resistance interpretation tool implemented in HBVdb, which identifies clinically validated resistance-associated mutations (RAMs) within the reverse transcriptase (RT) domain of the HBV polymerase gene [32]. This tool screens aligned RT sequences for substitutions associated with resistance to lamivudine, telbivudine, adefovir, entecavir, and tenofovir and classifies each sequence as drug sensitive or drug resistant on the basis of genotype-aware interpretation rules derived from published evidence.

## 5. Conclusions

This integrative evolutionary analysis suggests that the HBV genotype F is shaped by strong functional constraints on replication-associated proteins, coupled with adaptive flexibility in surface-exposed and regulatory regions. Functional interpretations derived from sequence analyses should be considered hypothesis-generating and warrant experimental validation in genotype F. Highly conserved glycosylation patterns and prevalent immune-related mutations highlight the importance of host–virus interactions in shaping genotype F evolution, whereas limited recombination preserves a largely tree-like evolutionary structure.

Although drug resistance mutations are limited, their presence follows canonical pathways and extends to recombinant genomes, underscoring the need for continued surveillance. Together, these findings provide a comprehensive genotype-specific framework for interpreting HBV-F diversity and emphasize the importance of incorporating evolutionary context into studies of viral fitness, immune escape, and the antiviral response.

## Figures and Tables

**Table 1:** Characterization of recombination events in HBV genotype F genomes.

	Site in X02763 *				Detection Methods ***						
Event	Begin	End	Recombinant Sequence(s)	Types **	RDP	Geneconv	Bootscan	Maxchi	Chimera	SiSscan	3Seq
1	487	1810	HE981179	F+G	3.45 × 10^−64^	5.48 × 10^−55^	1.31 × 10^−45^	1.93 × 10^−29^	1.22 × 10^−29^	1.81 × 10^−34^	4.13 × 10^−29^
2	1943	2786	JQ272887	G+F	8.30 × 10^−53^	4.54 × 10^−48^	1.23 × 10^−17^	3.92 × 10^−21^	2.21 × 10^−21^	3.76 × 10^−25^	1.69 × 10^−10^
			JQ272886	G+F							
3	2410	84	KJ586810	A+F	4.54 × 10^−52^	1.16 × 10^−44^	2.75 × 10^−54^	1.13 × 10^−19^	4.35 × 10^−25^	6.56 × 10^−32^	1.69 × 10^−10^
4	1810	2391	HE981180	G+F	8.94 × 10^−46^	9.31 × 10^−43^	9.71 × 10^−35^	2.99 × 10^−13^	2.02 × 10^−13^	8.86 × 10^−15^	1.69 × 10^−10^
5	2414	2788	KJ586811	D+F	3.98 × 10^−44^	7.25 × 10^−43^	1.46 × 10^−45^	3.56 × 10^−14^	4.47 × 10^−5^	5.24 × 10^−17^	1.69 × 10^−10^
6	641	1364	LT935669	F+D	4.33 × 10^−39^	2.93 × 10^−35^	1.27 × 10^−33^	7.89 × 10^−17^	4.52 × 10^−17^	1.07 × 10^−16^	1.69 × 10^−10^
7	1656	3209	KJ586803	A+B+D+F	5.29 × 10^−17^	1.26 × 10^−14^	2.35 × 10^−14^	8.22 × 10^−18^	9.92 × 10^−4^	6.45 × 10^−26^	8.27 × 10^−39^
8	1252	1670	JN688720	F+A	1.50 × 10^−35^	1.89 × 10^−29^	1.99 × 10^−22^	5.45 × 10^−10^	1.66 × 10^−10^	2.96 × 10^−8^	1.69 × 10^−10^
9	1596	2839	JQ707426	A+F+G	2.69 × 10^−26^	1.23 × 10^−21^	4.36 × 10^−25^	4.44 × 10^−18^	7.23 × 10^−19^	1.30 × 10^−32^	1.96 × 10^−34^
10	1552	1722	JQ272888	F+D	6.75 × 10^−28^	8.14 × 10^−23^	6.49 × 10^−21^	4.72 × 10^−6^	1.66 × 10^−6^	7.57 × 10^−8^	1.69 × 10^−10^
			AB214516	F+D							
11	1829	2099	HE981178	F+G	NS	6.21 × 10^−26^	3.19 × 10^−20^	5.19 × 10^−8^	5.15 × 10^−8^	4.43 × 10^−11^	1.69 × 10^−10^
			HE981177	F+G							
12	46	144	EF464097	F+G+H	7.86 × 10^−12^	5.69 × 10^−10^	1.03 × 10^−9^	2.32 × 10^−2^	NS	NS	5.13 × 10^−6^
			EF464098	F+G+H							
13	3216	290	MG098579	F+F	1.34 × 10^−6^	5.08 × 10^−9^	7.48 × 10^−6^	2.94 × 10^−3^	1.23 × 10^−3^	NS	9.94 × 10^−7^

* Positions are relative to the master reference strain X02763. ** HBV types are listed by major parent(s) followed by minor parent(s). *** NS = No significant *p* value was recorded for this recombination event via this method.

**Table 2:** Distribution and density of codons under selective pressure across HBV proteins and functional domains.

		Episodic Selection		Pervasive Selection		Negative Selection	
Protein (aa)	Domain (aa) *	#Codons **	Density ***	#Codons **	Density ***	#Codons **	Density ***
P (845)	TP (183)	19	0.10	9	0.05	76	0.42
	Spacer (165)	46	0.28	29	0.18	12	0.07
	RT (344)	18	0.05	7	0.02	121	0.35
	RNAse H (153)	4	0.03	2	0.01	54	0.35
S (400)	PreS1 (400)	51	0.13	22	0.06	84	0.21
	PreS2 (281)	38	0.14	20	0.07	38	0.14
	S (226)	31	0.14	18	0.08	24	0.11
C (183)	C (183)	28	0.15	22	0.12	62	0.34
X (154)	X (154)	82	0.53	16	0.10	15	0.10

* Domains: TP, terminal protein; RT, reverse transcriptase; RNase H, ribonuclease H; PreS1 and PreS2, presurface regions 1 and 2 of the surface protein, respectively; S, surface antigen; C, core protein; X, regulatory X protein. ** #Codons: absolute number of codon sites identified as under selection within each domain. *** Density: proportion of selected codons relative to the total number of codons in the domain.

**Table 3:** Distribution of predicted N-glycosylation sites across domains of the HBV genotype F large surface protein (L-HBsAg).

N-Glycosylation Site *	Domain	N-Glycosylation Counts **	Fraction
15 (4)	PreS1	362	1.000
37	PreS1	356	0.983
46	PreS1	19	0.052
123 (4)	PreS2	352	0.972
177	S	339	0.936
285	S	3	0.008
320 (146)	S	362	1.000
378	S	3	0.008

* L-HBsAg size: 400 amino acids (numbers in parentheses indicate the corresponding amino acid position in alternative HBsAg isoforms). ** Total dataset: 362 sequences.

**Table 4:** Distribution of amino acid substitutions within the major hydrophilic region (MHR) of the HBV surface antigen.

Subdomain (Positions)	Mutations (Counts) *
N-Term (100–123)	M103I (1), L110I (94), T118K (1), P120A (3), P120Q (3), K122R (3)
a″ determinant (124–147)	F134V (1), T140S (337), D144E (1)
C-Term (148–165)	F161Y (359), V164E (359)

* Counts: number of sequences in which the mutation was detected (total sequences = 362).

**Table 5:** Drug resistance–associated mutations in the HBV reverse transcriptase across HBV-F.

GenBank N°	Country	Subgenotype	Recombinant	Drug(s) Affected *	RT Mutations
FJ709457	Chile	F1b	No	LAM, LdT, ETV (I)	rtL180M + rtM204V
FJ709462	Chile	F1b	No	LAM, LdT, ETV (I)	rtL180M + rtM204V
HM585200	Chile	F1b	No	LAM, LdT, ETV	rtV173L + rtL180M + rtM204V
KP995116	Venezuela	F1b	No	LAM, LdT, ETV (I)	rtL180M + rtM204V
KC494401	Brazil	F2a	No	LAM, LdT, ETV (I)	rtL180M + rtM204V
KP995120	Venezuela	F2a	No	LAM, LdT, ETV (I)	rtL180M + rtM204V
HE974366	Martinique	F2b	No	LAM, LdT, ETV (I)	rtM204I
KP995118	Venezuela	F3	No	LAM, LdT, ETV (I)	rtM204I
EF464097	Brazil	F+G+H	Yes	LAM, LdT, ETV (I)	rtL180M + rtM204V
EF464098	Brazil	F+G+H	Yes	LAM, LdT, ETV (I)	rtL180M + rtM204V
JQ272888	Argentina	F+G	Yes	LAM, LdT, ETV (I)	rtL180M + rtM204V

* LAM: Lamivudine, LdT: Telbivudine, ETV: Entecavir. I: Intermediate (reduced susceptibility).

## Data Availability

All genomic data used in the present study were obtained from public databases (GenBank), together with their corresponding metadata. All relevant information is provided in the Supplementary Material (Table S1).

## Supplementary Materials

The following supporting information can be downloaded from https://www.mdpi.com/article/10.3390/ijms27052284/s1.
