# Supplementary figures and images for: From Genome Diversity to Inferred Functional Constraints: An Integrated Evolutionary Analysis of Hepatitis B Virus Genotype F
Source: Int J Mol Sci. 2026 Feb 28;27(5):2284. doi: 10.3390/ijms27052284 (PMC12985258; doi:10.3390/ijms27052284)

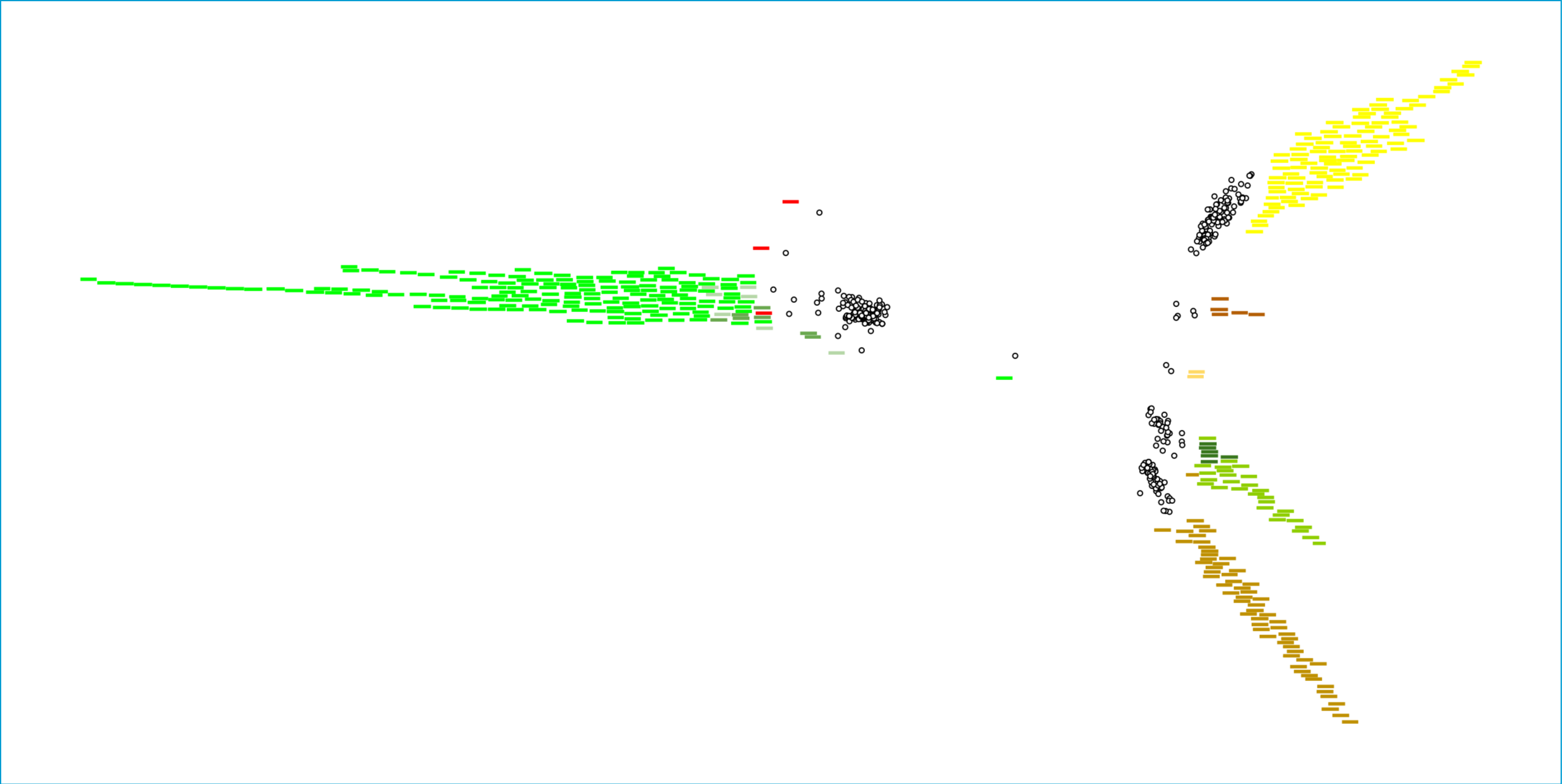

Supplement: Supplementary file 1 [file ijms-27-02284-s001.zip › Figure S1.png]
